# Another Chicken and Egg Story: Systematic Review on Lichen Planus as a Precursor for Celiac Disease in Adult Population

**DOI:** 10.7759/cureus.9526

**Published:** 2020-08-02

**Authors:** Sahar Khan, Shweta Patel, Saipavankumar M, Pousettef Hamid

**Affiliations:** 1 Internal Medicine, California Institute of Behavioral Neurosciences & Psychology, Fairfield, USA; 2 Psychiatry, California Institute of Behavioral Neurosciences & Psychology, Fairfield, USA; 3 Pediatrics, California Institute of Behavioral Neurosciences & Psychology, Fairfield, USA; 4 Neurology, California Institute of Behavioral Neurosciences & Psychology, Fairfield, USA

**Keywords:** celiac disease/complication, humans, prevalence, skin disease/ dermatology, etiology, lichen planus, autoimmune, gluten sensitivity, immunology, missed diagnosis

## Abstract

Celiac disease is receiving much attention due to the gluten-free diet trend. Many health-conscious individuals practice a gluten-free diet, even if they do not have celiac disease. As it is an autoimmune disorder, it is associated with many other autoimmune diseases. We were interested in one skin condition, another autoimmune disorder lichen planus as a correlative factor for celiac disease. The following systematic review may give some clues. We searched online resources including PubMed, PubMed Central, Cochrane library, and Google scholar for systematic reviews, traditional reviews, randomized controlled trials, and meta-analysis on celiac disease and lichen planus. We included human studies published in peer-reviewed journals in the English language. After reviewing 2389 initial results of our search, we excluded 1250 duplicates, 1108 abstracts, 42 irrelevant articles. We assessed the remaining 26 articles for their quality using various quality assessment tools.

After the quality assessment, we included nine final articles in our systematic review. Out of these nine studies, there were four systematic reviews, one traditional review, two case reports, and two observational studies. Only two articles had exclusively studied the specific association between celiac and lichen planus. The remaining studies included data that gave an overall association between other skin manifestations of celiac disease.

From our study, we could not establish the relationship between celiac disease and lichen planus. We need more case-control studies and clinical trials with a larger population to get conclusive data. From current data, we can conclude that both immunological processes correlate but there is no causation. There is also a need for clinical trials to explore the exacerbation of lichen planus due to celiac disease.

## Introduction and background

We saw a case of a 60-year-old patient discovered itchy skin lesions. Later, they diagnosed the lesions as lichen planus. He used multiple topical applications, including steroids, but no relief. Later he was diagnosed with celiac disease by endoscopy and biopsy. He noticed that removing gluten from his diet also improved his skin condition. The lesions were not itchy anymore, and with time and continued gluten-free diet, disappeared. He would advise fellow lichen planus patients to remove gluten from the diet and see the results. He would refer to it as “the gluten connection.”

Celiac disease is an autoimmune disorder affecting 1% of the population [1]. Importance of wheat intake to gluten sensitivity was first identified in children by physician William Dicke [2]. Research on vaccination for celiac disease was discontinued due to unsatisfactory results and the only available treatment today is a gluten-free diet for life [3]. In 1956, Margot Shiner developed biopsy tube for the small intestine [4]. Human leukocyte antigen (HLA) studies in monozygotic twins suggest genetic components. Through more research, scientists discovered circulating antibodies which suggested immunological mechanisms. Several other immunological mechanisms believed to coexist with celiac disease such as diabetes mellitus, autoimmune thyroid disease, and diagnostic cutaneous manifestation dermatitis herpetiformis [5]. Marks et al. established association of dermatitis herpetiformis with celiac disease in 1965 and 1968 [6].

Another immunologic disease studied is lichen planus. The overall estimated pooled prevalence of oral lichen planus (OLP) was 0.89% among the general population and 0.98% among patients [7]. A higher prevalence of OLP was present in non-Asian countries, among women, and 40 years and older. We should consider these findings with caution because of the high heterogeneity of the included studies [7]. It can present as a characteristic lacy pattern of distribution called Wickham's striae. We can diagnose it by punch biopsy of 4 mm diameter. It can range from mild to severe. Treatment for severe lichen planus involves systemic tretinoin. It can occur in the skin and mucous membranes. Lichen planus is associated with a few autoimmune disorders and infectious diseases such as hepatitis C.

What we know so far is that both the diseases are immunological processes. Though both the above diseases are extensively studied separately, the association of the diseases remains vague. The purpose of these reviews is to look up other systematic reviews and discuss any clear correlation between the two diseases. If we establish a connection between the two, early detections of lichen planus and subsequent testing for the celiac disease may make patients go gluten-free, thus reducing the damage to the intestine.

## Review

Methodology and results

**Figure 1:**
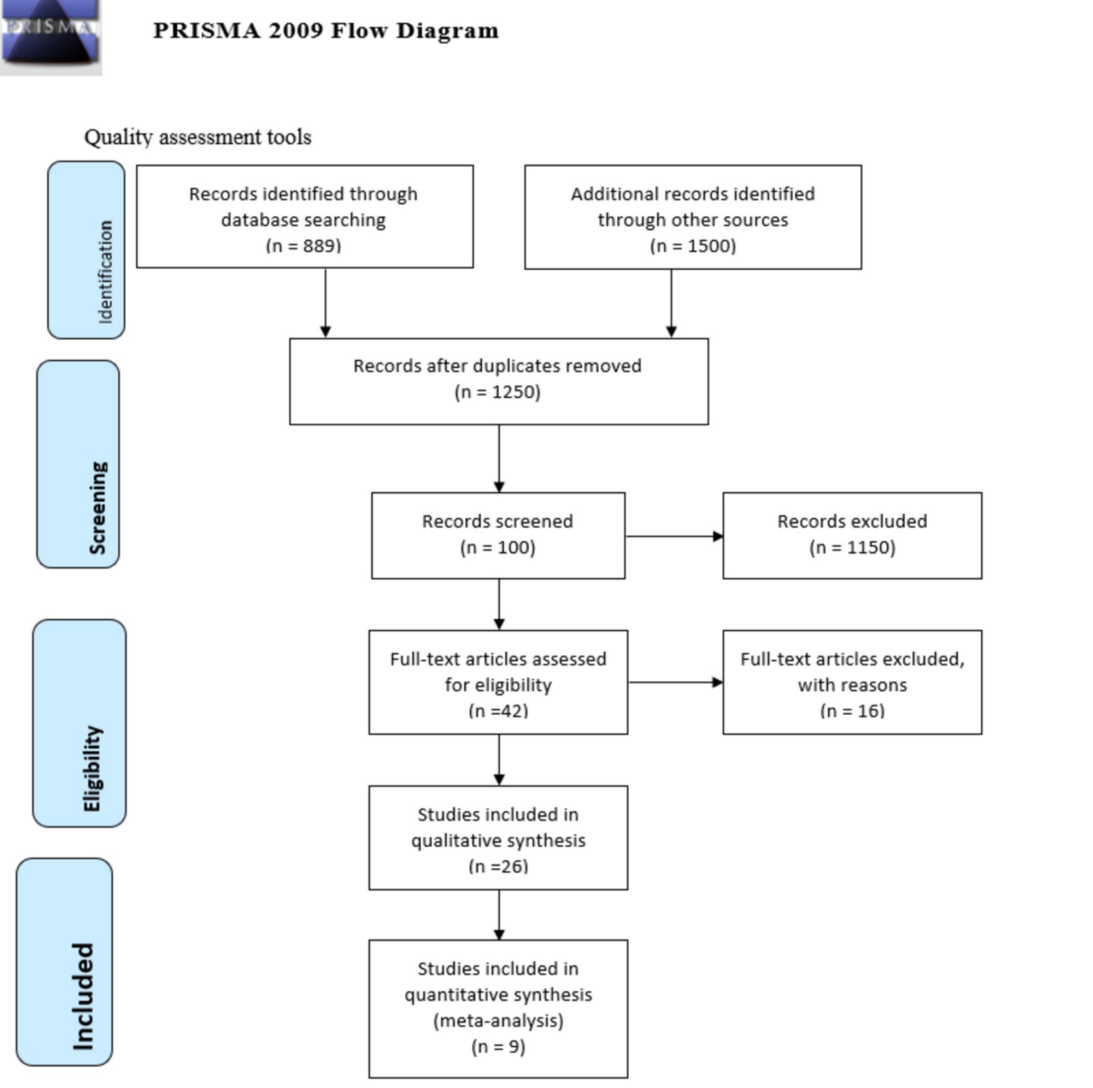
Prisma flow diagram for correlation of celiac disease and lichen planus

Following the PRISMA protocol as seen in Figure 1, we selected multiple online databases [8]. These were PubMed, PubMed Central, Google Scholar, Cochrane Library.

Search strategy

We reviewed abstract type articles and other articles which were relevant to the topic of discussion. These included PubMed, Cochrane review, Web of Science Google Scholar.

Types of study

We used mixed types of studies, which included clinical trials, systematic reviews. All articles studied were peer-reviewed. All articles were ethical and legal.

MeSh keywords included celiac disease/complication, humans, prevalence, skin disease/dermatology, etiology, lichen planus, autoimmune, gluten sensitivity, immunology.

Inclusion criteria

Articles were selected without a time frame to identify the first-ever connection between the above-said processes. We analyzed articles mentioning lichen planus (cutaneous and oral) and celiac disease, gluten sensitivity, gluten allergy and allergy to wheat germ. Only adult human studies were reviewed. We included articles from all over the world, written in the English language.

Exclusion criteria

We excluded pediatric and animal studied and articles in languages other than the English language. We also excluded similar diseases such as lichen sclerosis and Crohn's disease (CD).

Search strategy is summarized in Table 1 below.

**Table 1:** Search strategy for our systematic review

MeSH Keywords	Database	Search Results
Celiac disease	PubMed	26921
	Google Scholar/Others	1500
Oral lichen planus	Pub Med	9903
	Google Scholar/Others	70,100
Immunology	Pub Med	4114
	Google Scholar/Others	7828
Extraintestinal	Pub Med	1648
	Google Scholar/Others	11500
Dermatological	Pub Med	301
	Google Scholar/Others	15400
Autoimmunity	Pub Med	3087
	Google Scholar/Others	47200

Results

After the initial screening, we assessed 42 articles for eligibility and excluded 16 articles that did not fit the main title of the analysis. We divided the remaining 26 articles according to the type of study and applied appropriate qualitative analysis tools.

We used different tools to determine the quality of each article. These were the SANRA tool for a narrative article or traditional review [9], AMSTAR for systematic review [10], Cochrane risk of bias tool for randomized controlled trials (RCT) [11]. The objective of the quality assessment was to establish a clear relationship between celiac disease and lichen planus if any.

We included total nine articles. As seen in Table 2, we included two case reports, two observational studies, four systematic reviews, one traditional review, and no meta-analysis.

**Table 2:** Summary of articles reviewed

Year	Type of study	Author	Journal	Association present Y/N	Population
1941	Case report	Hamilton Newman [12]	Canadian Medical Association Journal	Y	Adult Caucasian
1993	Case report	Fortune and Buchanan [13]	The Lancet	Y	Adult Caucasian
2006	Systematic review	Abenavoli et al. [14]	World Journal of Gastroenterology	Y	Adult Caucasian
2006	Observational retrospective study	Jones et al. [15]	Nutrition Journal	N	Adult Caucasian
2009	Systematic review	Abenavoli et al. [16]	Expert Reviews	Y	Adult Caucasian
2014	Observational retrospective study	Cigic et al. [17]	PubMed/Springer	Y	Adult Caucasian
2014	Traditional review	Compilato et al. [18]	Journal of the European Academy of Dermatology and Venereology	Y	Adult Caucasian
2018	Review	Rodrigo et al. [19]	Nutrients	Y	Adult Caucasian
2019	Systematic review	Abenavoli et al. [20]	Medicina	Y	Adult Caucasian

Discussion

We found the first-ever written evidence of the association described in a Canadian journal as a case report in 1941 by Hamilton Newman [12]. The case described a 47-year-old man who developed an extreme irritation of the skin in the spring of 1939. Affected areas were palms wrists and arms as far as elbow on the flexures surfaces of the skin, which had papules, and some were scaly. On the right flank, there was a patch affecting the skin. At first, the color of the affected skin was red blue but later evolved to form characteristic lichen planus appearance. However, despite treatment, there was no improvement in the rash. Further questioning revealed that the patient had been taking a wheat germ preparation. When he stopped the wheat germ and observed the patient after six weeks, the eruption had disappeared, and symptoms improved. Whenever the patient took wheat germ in any form such as oil, there was an immediate flare-up of the eruptions.

A 1999 study by Ventura et al. showed an increased prevalence of the autoimmune disease among 909 patients who had celiac disease when compared with controls [21]. However, there was no significant difference compared with 163 Crohn's patients. Logistic regression analysis showed that the age at celiac disease diagnosis was an essential factor of autoimmune disease later in life. Sategna Guidetti et al. demonstrated that the duration of gluten exposure is a predictor of autoimmune disease [22]. Cataldo and Marino have demonstrated a higher prevalence of the autoimmune disease among first-degree relatives of children with celiac disease (4.8%) when compared with first-degree relatives of healthy children (0.86%) [23].

They stated this increased risk had to do with the higher prevalence of the silent celiac disease. It is unclear whether untreated celiac disease is responsible for the increased occurrence of autoimmune disease. However, it is worth the clinician to be aware of these associations. Patients with a newly diagnosed celiac disease should know that their risk of developing another autoimmune disease cannot significantly increase after diagnosis and treatment.

The next case report was Fortune and Buchanan’s article in Lancet about 70-year-old nonsmoker male of increasing oral soreness and eczema with no history of gastrointestinal symptoms [13]. Oral examination revealed lichen planus confirmed on oral biopsy. The patient started corticosteroid mouthwashes. Blood tests showed iron deficiency anemia, as well as deficiency of vitamin B12 and folate. A jejunal biopsy demonstrated villous atrophy and diagnosed the patient with celiac disease. Oral soreness and anemia improved within six months after a gluten-free diet. Symptoms exacerbated when the patient took gluten. However, there was no concrete finding whether oral lesions were a manifestation of gluten enteropathy or the malabsorptive effect of rapidly dividing mucosal cells already predisposed to soreness by pre-existing lichen planus.

By 2006, Abenavoli et al. did a systematic review of skin manifestation associated with celiac disease. According to this review, the data for celiac disease and cutaneous disorders was not homogeneous and only based on case reports. Therefore, they grouped all reviews done.

In the subsection of Oral Lichen Planus, they hinted at the association between CD and persistent oral ulceration, glossitis, angular cheilitis, and burning mouth.

Jones et al. in 2006 conducted an observational retrospective study in a rural setting exploring 'silent celiac disease' [15]. The study included 70 celiac patients who had first presented with symptoms different from the classic presentation. They found that a dermatologist referred 22% of the patients with the chief complaint of skin rash, which was a manifestation of celiac disease after a complete investigation.

Abenavoli et al. conducted a review of the various cutaneous manifestation of celiac disease in 2009 [16]. However, they again stressed that data was not homogeneous and needed for the controlled study to find conclusive evidence of the involvement of the said autoimmune diseases.

This query answered in 2014, where a study was published that exclusively mentioned lichen planus and celiac disease by Cigic et al. [17]. The study quoted Rashid et al. that oral lichen planus is a possible manifestation [24]. No data are available on the prevalence of CD in patients with OLP. This study aimed to investigate the prevalence of CD, CD marker antibodies, and HLA-DQ in patients with two different forms of OLP (reticular and erosive) and to compare the results with matched healthy controls from the population [17]. Their analysis found eight new CD cases. The predominance of CD was 14.28% in OLP patients, compared to 0% in matched healthy controls and overall population 1-2%. IgA tTG is a sound screening system built to diagnose CD. Only three patients with OLP (5.36%) gastrointestinal symptoms were present in 137 patients.

The other article in the discussion is a traditional review done by Compilato et al., which also exclusively mentioned lichen planus and celiac disease [18]. They concluded that improved nutritional status leads to relief in oral soreness as opposed to the often less successful palliative medications. Therefore, it was suggested to search for possible nutritional deficiencies in any patient with atrophic/erosive oral lesions, including OLP, and subsequently to include screening for CD.

Rodrigo briefly mentioned in their article in 2018 about lichen planus as one of the many cutaneous manifestations of celiac disease [19]. They discussed the strength of evidence of various skin manifestations with celiac disease. However, they briefly mentioned lichen planus as cited in other articles, and no statistical data provided.

The last systematic review we analyzed was of Abenavoli et al. [20]. The detailed review included evidence-based studies. Lichen planus was a class 3B evidence as a result of an individual case-controlled study [20].

Limitations of the research

There was some limitation during the research project. One of the limitations was that not many articles solely focused on the two diseases in question. Few of the studies were inconclusive and were discontinued due to a lack of funding. Also, some articles referenced were no longer available. Due to the outbreak of COVID-19, we could only search online as libraries were closed, so a manual search was not possible. Furthermore, almost all the studies mentioned we found were on the adult Caucasian population; thus, a more diverse aspect of this study could not occur.

## Conclusions

Often, the skin manifestations occur before or together with the gastrointestinal pathology. Hence dermatologists and gastroenterologists can detect various cutaneous signals so that they can diagnose at the earliest. Since celiac disease is silent in most of the patients, those with obvious symptoms of mouth ulcers and skin eruptions should be tested for celiac disease. Therefore, if they start a gluten-free diet earlier the symptoms of celiac disease as well as lichen planus can be controlled and lead to a better quality of life for these individuals.
